# Sirolimus-induced pneumonitis

**DOI:** 10.4103/0971-4065.53329

**Published:** 2009-04

**Authors:** U. Singh, A. Gupta, S. Jasuja

**Affiliations:** Indraprastha Apollo Hospital, Sarita Vihar, Mathura Road, New Delhi - 110 076, India

A 15 year-old girl with reflux nephropathy ESKD underwent a related renal transplant in March 2000. Initial immunoupprressants administered to the patient included cyclosporine, azathioprine, and prednisolone. In January 2003, she had acute allograft dysfunction due to CNI toxicity with chronic allograft nephropathy (CAN) detected by biopsy. Thus, cyclosporine and azathioprine were withdrawn and replaced with rapamycin and mycophenolate mofetil. The serum creatinine level at the time of the change was 2.2 mg/dL, urine routine microscopy showed Trace proteinuria. Sirolimus levels were monitored and kept between 5 and 10 ng/mL. With the withdrawal of cyclosporine, renal parameters showed significant improvement and the serum creatinine level stabilized at 1.6 mg/dL.

**Figure 1:**
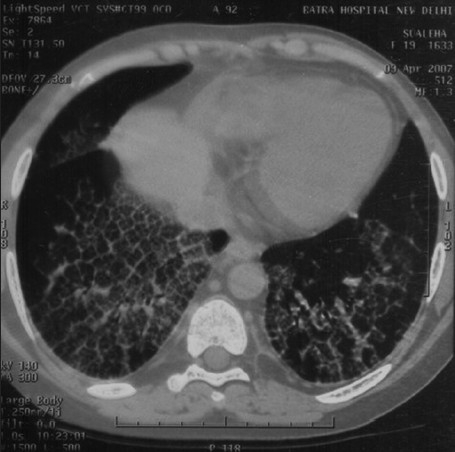
Sirolimus-induced pneumonitis

**Figure 2:**
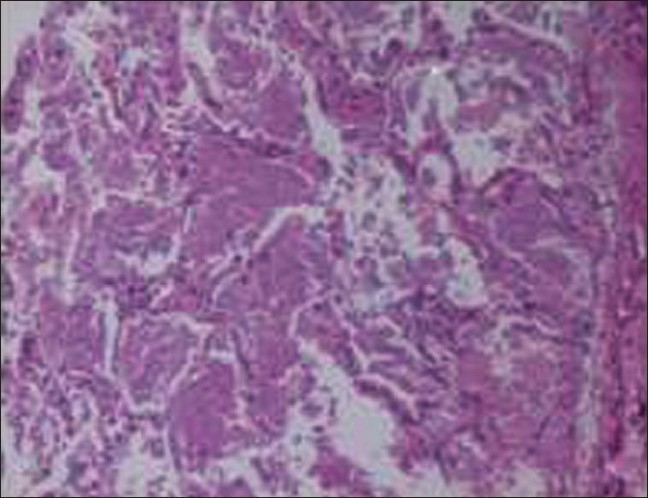
Lung biopsy showing parenchymal lung lesion. Transbronchial lung biopsy shows normal bronchial epithelium, bronchial cartilage, and surrounding lung tissue. Alveoli contain a granular proteinaceous material (arrows). This material is also seen lying loosely in the extraalvelor area. No fungus, acid-fast bacillus, or pneumocystis carinii are seen.

**Figure 3:**
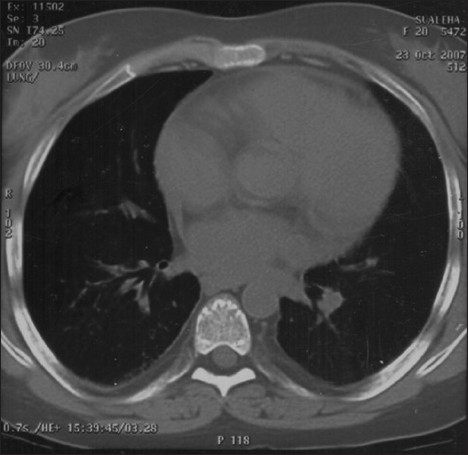
Resolved Sirolimus-induced pneumonitis

In April 2007, after approximately four years of the change, the patient presented with progressive breathlessness without any other systemic manifestations or expectoration. Computerised tomographic (CT) of the chest showed bilateral, mosaic ground-glass opacification and thickened interlobular septae [Figure 1]. Bronchoscopy, broncheoalveolar lavage, and pulmonary biopsy excluded infective etiology such as pneumocystis carnii, pulmonary Koch's, and fungal pneumonia [Figure 2]. By exclusion, the etiology was narrowed down to rapamycin-induced parenchymal lung lesions. Thus, rapamycin was withdrawn and the patient was serially followed on two drugs, mycophenolate mofetil and prednesolone. She improved clinically in twelve weeks and radiologically over six months. The followup CT of the chest showed significant clearing of the reticular and ground glass opacities [Figure 3].

Sirolimus (rapamycin) is an immunosuppressive agent that was approved by the Food and Drug Administration (FDA) in 1999 for use in renal transplantation. The macrocyclic triene antibiotic is produced by the actinomycete, *Streptomyces hygroscopicus*. Sirolimus acts by arresting the progression of cells from the G_1_ phase to the S phase by interaction with at least two intracellular proteins. Forty-three cases of pulmonary toxicity due to Sirolimus (rapamycin) have been reported including cases of interstitial pneumonitis, fibrosing alveolitis, and pulmonary hemorrhages.[1] The majority of these cases have occurred in renal transplant recipients within the initial six months of iniciation of the drug and lesions have generally responded to the lowering or withdrawal of the drug dosage. Most of the patients present with progressive breathlessness, weakness, cough, and bilateral lower zone pulmonary opacities, and over half of the cases present with fever. Our patient fulfilled all these criteria, except for the delayed presentation. The mechanism of pulmonary toxicity of Sirolimus (rapamycin) is not clear; direct toxicity, immunomediated toxicity, or both have been proposed as the mechanism.[1] The presence of lymphocytes in lung biopsies suggests an immunomediated mechanism. Possibly, some cryptic pulmonary antigens induce an autoimmune response. However, the rapid clearance of lung lesions with the discontinuation of the drugor dose reduction without steroids, favors direct toxicity.
